# Study of parasitic resistance effects in nanowire and nanoribbon biosensors

**DOI:** 10.1186/s11671-015-0794-6

**Published:** 2015-02-21

**Authors:** Ioannis Zeimpekis, Kai Sun, Chunxiao Hu, Owain Thomas, Maurits RR de Planque, Harold MH Chong, Hywel Morgan, Peter Ashburn

**Affiliations:** Zepler Institute, School of Electronics & Computer Science, University of Southampton, Southampton, SO17 1BJ UK; Oxford Instruments Plasma Technology, Yatton, Bristol, BS49 4AP UK

**Keywords:** Biosensor, Nanowire, Nanoribbon, Parasitic resistance, Differential biosensor, pH sensor

## Abstract

In this work, we investigate sensor design approaches for eliminating the effects of parasitic resistance in nanowire and nanoribbon biosensors. Measurements of pH with polysilicon nanoribbon biosensors are used to demonstrate a reduction in sensitivity as the sensor length is reduced. The sensitivity (normalised conductance change) is reduced from 11% to 5.5% for a pH change from 9 to 3 as the sensing window length is reduced from 51 to 11 μm. These results are interpreted using a simple empirical model, which is also used to demonstrate how the sensitivity degradation can be alleviated by a suitable choice of sensor window length. Furthermore, a differential sensor design is proposed that eliminates the detrimental effects of parasitic resistance. Measurements on the differential sensor give a sensitivity of 15%, which is in good agreement with the predicted maximum sensitivity obtained from modeling.

## Background

Over the past 40 years, Ion Sensitive Field Effect Transistors (ISFETs) have been widely researched for applications as ion [1,2], pH [3], and protein sensors [4]. More recently, nanowire and nanoribbon biosensors [5,6] have been developed as improved devices because their high surface-to-volume ratio gives high sensitivity. Nanowires can be fabricated using bottom-up [7] or top-down [8-13] processes. The bottom-up approach has the advantage of simplicity, but there is no precise control of nanowire size and position, and this makes it difficult to achieve low resistance ohmic contacts. Top-down approaches overcome these shortcomings and can be based around silicon CMOS [14] or thin-film transistor (TFT) [15] technologies. Both approaches are compatible with mass manufacture and allow heavily doped contact regions to be incorporated to reduce contact resistance. However, the cost of a biosensor is determined by the number of steps in the manufacturing process, so the inclusion of heavily doped contacts increases cost.

The sensitivity of a biosensor is defined as the relative change in conductance after attachment of the target biomolecule and is inversely proportional to the doping concentration in the nanowire or nanoribbon [16]. Consequently, biosensors that are designed for high sensitivity have a relatively large resistivity, and high values of contact resistance are likely unless additional heavily doped contact regions are incorporated into the manufacturing process. Moreover, to accommodate measurements in a liquid environment, biosensor metallization and contact regions are covered with a passivation layer to protect them from the analyte solution. The passivated regions are not influenced by the attachment of target biomolecules to the surface of the sensor and therefore act as a parasitic resistance. When protein sensing is performed, the relative current changes can be rather small for some proteins [15]. In this situation, signal read-out considerations require sensor operation at higher currents, where the effects of parasitic resistance can be important. To date, there have been no published studies of the effect of parasitic resistance on the performance of nanowire or nanoribbon biosensors.

The aim of this work is to investigate the effects of parasitic resistance on biosensor sensitivity. Thin-film polysilicon nanoribbon biosensors are fabricated with different sensing window lengths, and it is shown that the sensitivity is lower for shorter sensing windows. These results are interpreted using a simple empirical model that relates biosensor sensitivity to parasitic resistance and sensing window length. Finally, a differential biosensor design is demonstrated that eliminates the detrimental effects of parasitic resistance and delivers a value of sensitivity equal to the maximum predicted by the model. This high value of sensitivity is achieved without the need to incorporate heavily doped contact regions.

## Methods

Polysilicon nanoribbon biosensors were fabricated with different channel lengths using the TFT process detailed in our previous work [6] and measured in dry and wet ambient. Electrical characterization was performed using an Agilent B1500A I/V-based probe-station (Agilent Technologies Singapore (International) Pte. Ltd., Singapore). The sensors were measured in a Faraday cage enclosure box to minimize interference. For the characterization of the sensors, electrical contact was made on the TiN electrodes using Cascade micropositioner probes. The backside of the substrate was grounded through the probe-station chuck to prevent biasing of the channel through it. Transmission line measurements (TLMs) were performed on test structures, and values of sheet resistance (*R*_s,dry_) and contact resistance (*R*_c_) were extracted to be 448 kΩ/sq and 447 kΩ, respectively. From these values, the total parasitic resistance (*R*_par_) was calculated to be 1.6 MΩ. For wet sensing with pH buffers, a liquid gate was formed by pipetting liquid in the sensing window while a potential was applied using a Ag/AgCl electrode immersed in the solution. pH measurements were carried out using universal buffer mixture (UBM) solutions of pH 3, 5, 7, and 9. Those measurements were initiated by introducing pH 9 in the sensor window while applying a 50 mV bias to a Ag/AgCl electrode and a channel potential of 100 mV. After a 10 min stabilization time, the measurement was reset and the buffers were introduced sequentially from high to low pH and back to high pH to complete a full titration. All sensing measurements were performed at the same sensor biasing point so that values of sensitivity could be quantitatively compared.

## Results and discussion

**Figure 1:**
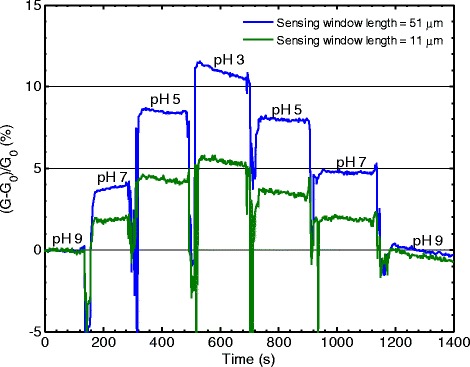
**Normalized conductance change (sensitivity) measured after changes of pH for two sensors with different sensing window lengths.**

Figure 1 shows the normalized conductance change as a function of time when the biosensor is exposed to different pH buffers for two different TFT biosensors with sensing window lengths of 51 and 11 μm. For the 51 μm window sensor, the conductance increases by approximately 11% for a pH change of 6 units, from pH 9 to pH 3. A subsequent increase in pH results in a decrease of conductance back to the original value. A similar, though smaller, trend is observed for the sensor with the shorter sensing window of 11 μm with a conductance increase of 5.5% from pH 9 to pH 3. Therefore, the longer 51 μm sensor has a higher sensitivity than the shorter 11 μm sensor. This indicates a relation between sensitivity and sensing window length.

**Figure 2:**
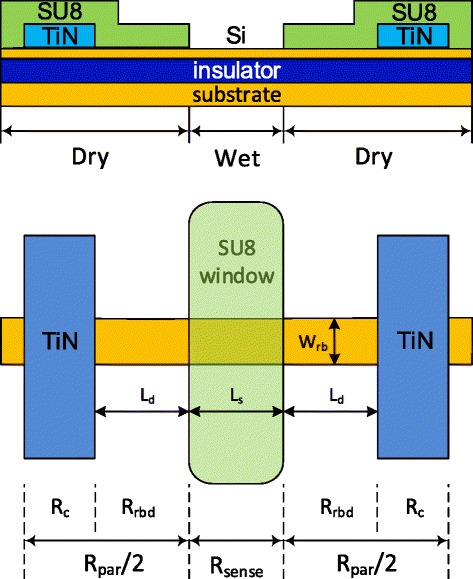
**Schematic cross-section and plan views of the polysilicon nanoribbon biosensor.**

To study the relationship between sensitivity and sensor dimensions, a simple model is presented in Figure 2. The model includes three sections; the wet section (*R*_sense_) where the sensor is exposed to liquid and two dry sections (*R*_par_) where it is protected from liquid by SU8. All sections contribute to the overall resistance (*R*), which can be measured directly from electrical measurements. The dry section includes the contact resistance from the TiN/Si contacts (*R*_c_) and the parasitic resistance of the nanoribbon region covered by SU8 (*R*_rbd_).

The total resistance of the biosensor is:

1 $$ \begin{array}{l}R={R}_{\mathrm{s}\mathrm{ense}}+{R}_{\mathrm{par}}={R}_{\mathrm{s}\mathrm{ense}}+2{R}_{\mathrm{rb}\mathrm{d}}+2{R}_{\mathrm{c}}\\ {}\kern1.44em =\frac{R_{\mathrm{s},\mathrm{wet}}{L}_{\mathrm{s}}}{W_{\mathrm{rb}}}+2\frac{R_{\mathrm{s},\mathrm{dry}}{L}_{\mathrm{d}}}{W_{\mathrm{rb}}}+2{R}_{\mathrm{c}}\end{array} $$

where *R*_s_,wet and *R*_s,dry_ are the sheet resistances of the wet and dry nanoribbon regions, *L*_s_ and *L*_d_ are the lengths of the wet and dry nanoribbon regions, *W*_rb_ is the nanoribbon width, and *R*_c_ is the contact resistance of the TiN/Si contacts. Here, *R*_s,dry_ and *R*_c_ can be measured using TLM test structures. The dimensions of the device, *L*_s_, *L*_d_, and *W*_rb_, can be measured optically. Using (1), *R*_s,wet_ can be calculated from the measured biosensor resistance, *R*. The sensitivity is defined as the normalized conductance change and is given by:

2 $$ \mathrm{Sensitivity}=\frac{G-{G}_0}{G_0}=\frac{1/R-1/{R}_0}{1/{R}_0} $$

where *R*_0_ and *G*_0_ are the resistance and the conductance at a reference level (pH 9 in this work), respectively.

**Figure 3:**
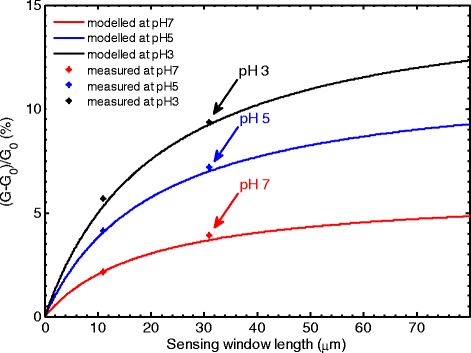
**Modeled normalized conductance change (sensitivity) as a function of sensing window length at different values of pH.** The graph also includes measured data from two devices with sensing window lengths of 11 and 31 μm.

Figure 3 shows the modeled sensitivity as a function of sensing window length (solid lines) calculated with parameters obtained from measurements on a sensor with a sensing window length of 51 μm. The measured sensitivities of two devices with sensing window lengths of 11 and 31 μm are also presented (points). The curves closely match the measured data indicating that the experimental data can be modeled with reasonable accuracy.

**Figure 4:**
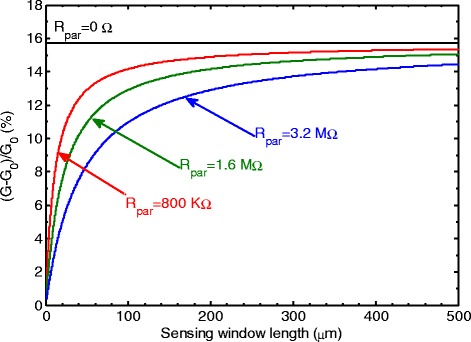
**Predicted normalized conductance change as a function of window length for different values of parasitic resistance.**

To investigate how practical sensors can be designed for maximum sensitivity, the normalized conductance change at pH 3 is plotted in Figure 4 over a wide range of sensing window lengths. The green line in Figure 4 is plotted using measured data from the fabricated sensors and used a parasitic resistance of 1.6 MΩ. For comparison, the red and blue lines show predictions for different values of parasitic resistance of 800 kΩ and 3.2 MΩ, respectively. For an ideal sensor, with a parasitic resistance of zero, the sensitivity remains constant with a value of 15.6%. In contrast, for the three sensors with parasitic resistance, the sensitivity approaches the ideal value at long channel lengths. In order to achieve a sensitivity of 90% of the maximum (14.1%), biosensor lengths of 85, 185, or 380 μm are required for values of parasitic resistance of 800 kΩ, 1.6 MΩ, and 3.2 MΩ, respectively. This result shows that to approach the maximum value of sensitivity, biosensors with long lengths must be used. This conclusion contradicts the model presented in [16], which predicted that the biosensor sensitivity increased for shorter channel lengths. However, the model presented in [16] did not include the effects of parasitic resistance and hence predicts the behavior of idealised, rather than practical, biosensors.

**Figure 5:**
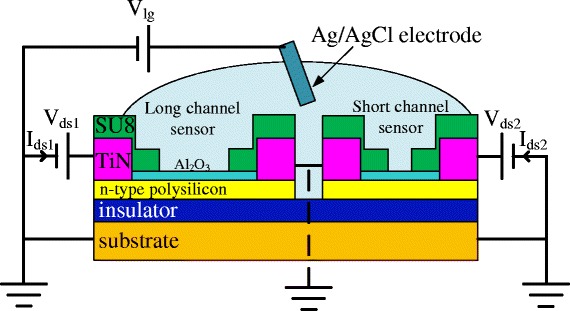
**Schematic illustration of the differential design and measurement configuration.** Two sensors are used for the differential measurement, with long and short sensing windows. The sensors are otherwise identical.

To maximize the sensitivity, a differential biosensor design is proposed in Figure 5, which measures the difference in resistance between the two biosensors with different sensor window lengths. As the two biosensors have equal values of parasitic resistance (*R*_par1_* = R*_par2_) but different window lengths (*R*_sense1_ ≠ *R*_sense2_), subtraction eliminates the parasitic resistance from the equation. This differential sensor design therefore should eliminate the parasitic resistance effects and give the maximum value of sensitivity.

**Figure 6:**
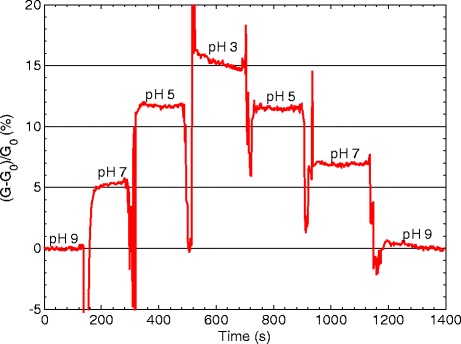
**Measured normalized differential conductance change (sensitivity) for the differential sensor after changes in solution pH.**

Figure 6 shows the measured normalized conductance change as a function of sensor window length for the differential sensor. The conductance change follows a similar trend to the results in Figure 1 for the individual sensors, but the change is larger. The normalized conductance change between pH 9 and pH 3 is 15%. This is a 37% improvement over the result from the 51-μm-long sensor and a 174% over the 11 μm sensor.

Table 1 compares the measured values of sensitivity obtained using the differential sensor design with predicted maximum values of sensitivity obtained from the model results in Figure 3. The differentially measured values of sensitivity are in excellent agreement with the predicted maximum values of sensitivity for all pH values. It can consequently be concluded that the differential sensor design presented here has eliminated the detrimental effects of parasitic resistance and delivered the maximum value of sensitivity that is possible for a given nanoribbon doping concentration.

**Table 1:** **Comparison of measured sensitivity of the differential biosensor with modeled maximum values (for parasitic resistance equal to zero)**

**pH value**	**Differentially measured sensitivity**	**Predicted maximum sensitivity**
7	6.0%	6.0%
5	11.5%	11.6%
3	15.1%	15.6%

The sensing window lengths of the devices measured in this work were chosen so that the performance of the individual sensors in the differential pair could be separately demonstrated. The requirement for the differential sensor to work is that both sensors have the same parasitic resistance but different sensing window lengths. In practice, a smaller differential biosensor footprint could be achieved if the sensing window length of one sensor was set to zero. When performing the differential measurement, the zero length sensor would then cancel the parasitic resistance of the finite length sensor. This approach allows the differential sensor design to be miniaturized, while maintaining the maximum sensitivity.

## Conclusions

This paper has studied the effect of parasitic resistance on biosensor sensitivity. The results show that a high value of parasitic resistance reduces the sensitivity of the sensor, while a long sensing window reduces this sensitivity degradation. A model has been formulated to describe this relationship and used to identify how the sensing window length can be chosen to minimize the negative effects of parasitic resistance. Furthermore, a differential biosensor design has been proposed, and the measurements have indicated that the deleterious effects of parasitic resistance have been eliminated.
